# A clinical perspective on plasma cell leukemia; current status and future directions

**DOI:** 10.1038/s41408-021-00414-6

**Published:** 2021-02-04

**Authors:** Sherilyn A. Tuazon, Leona A. Holmberg, Omar Nadeem, Paul G. Richardson

**Affiliations:** 1 Clinical Research Division, Fred Hutchinson Cancer Research Center, Seattle, WA USA; 2 Department of Medicine, Medical Oncology, University of Washington, Seattle, WA USA; 3 Dana-Farber Cancer Institute, Boston, MA USA; 4 Harvard Medical School, Boston, MA USA

**Keywords:** Myeloma, Myeloma

## Abstract

Primary plasma cell leukemia (pPCL) is an aggressive plasma cell disorder with a guarded prognosis. The diagnosis is confirmed when peripheral blood plasma cells (PCs) exceed 20% of white blood cells or 2000/μL. Emerging data demonstrates that patients with lower levels of circulating (PCs) have the same adverse prognosis, challenging the clinical disease definition, but supporting the adverse impact of circulating PCs. The cornerstone of treatment consists of combination therapy incorporating a proteasome inhibitor, an immunomodulatory agent, steroids, and/or anthracyclines and alkylators as part of more-intensive chemotherapy, followed by consolidative autologous hematopoietic cell transplantation in eligible patients and then maintenance therapy. Monoclonal antibodies are also currently being evaluated in this setting with a strong rationale for their use based on their activity in multiple myeloma (MM). Due to limited therapeutic studies specifically evaluating pPCL, patients with pPCL should be considered for clinical trials. In contrast to MM, the outcomes of patients with pPCL have only modestly improved with novel therapies, and secondary PCL arising from MM in particular is associated with a dismal outlook. Newer drug combinations, immunotherapy, and cellular therapy are under investigation, and these approaches hopefully will demonstrate efficacy to improve the prognosis of pPCL.

## Introduction

Plasma cell leukemia (PCL) is the rarest yet most aggressive plasma cell disorder. PCL was originally defined by an absolute plasma cell (PC) count greater than 2 × 10^9^/L in the peripheral blood (PB) and more than 20% circulating PCs^1^. At present, only one of the original two requirements is used to define PCL according to the criteria by the World Health Organization and the International Myeloma Working Group (IMWG)^2,3^. The definition of PCL is currently an area of controversy as recent studies suggest that the presence of ≥5% circulating abnormal PCs but not meeting the 20% cutoff have a similar adverse prognostic impact and poor survival as patients with >20% abnormal PCs^4,5^. Therefore, patients with PCs comprising ≥5% of the PB white cell differential count may be considered as having PCL and can be treated as such. Another limitation of the current diagnostic definition of PCL is that it does not take into consideration plasma cell clonality, which can be characterized using multiparametric flow cytometry^6^. Establishing the presence of a malignant clonal population is important as reactive plasmacytosis can occur due to a variety of infections, neoplastic or inflammatory conditions^7–10^.

PCL is referred to as primary when the leukemic phase presents at diagnosis, or secondary when leukemic progression occurs in the context of preexisting multiple myeloma (MM). Historically, 60–70% of cases have been reported to be primary PCL (pPCL), whereas ~40% are secondary^11^. In recent years, there has been an increase in the incidence of secondary PCL^12^, likely related to more effective therapies contributing to both improved survival and clonal selection over time. In this review, we will focus on pPCL, and also comment on secondary PCL, when applicable.

## Clinical and laboratory manifestations of PCL

In comparison to MM, pPCL has distinct biological and clinical features. Because pPCL is rare, data has been primarily obtained from retrospective studies with small numbers of patients. The median age at diagnosis of pPCL is 61 years, approximately 10 years younger than the average age of diagnosis of a typical MM patient^13^. pPCL is characterized by significant anemia, thrombocytopenia, renal insufficiency, hypercalcemia, and increased tumor burden (reflected by elevated lactate dehydrogenase (LDH) and β2-microglobulin, as well as marked bone marrow (BM) plasma cell infiltration^2,14^. Light chain and nonsecretory subtypes are more commonly observed in pPCL^12^. Extramedullary involvement is similarly more common in pPCL, and may be related to tumor cells having reduced expression of adhesion molecules (CD56, LFA-1, LFA-3, VLA-5), which impair retention of PCs within the BM^15–18^. Conversely, lytic bone lesions are less common in pPCL than in MM^12^.

The immunophenotype of MM and pPCL express both CD38 and CD138; however, pPCL cells have higher expression of CD20, CD27, CD28, and CD45 and lower expression of CD9, CD56, CD117, and HLA-DR compared to MM^19–21^. The karyotype of pPCL frequently demonstrates hypodiploidy, associated with poor prognosis^22,23^. Chromosome 1 aberrations (deletion 1p and 1q amplification), now considered adverse cytogenetic abnormalities by the IMWG in MM^24^, are also more frequent in pPCL^25,26^. Importantly, deletion 17p, a notoriously high-risk marker, is observed in up to 50% of pPCL^12^. The most common abnormality in pPCL is t(11;14) with a frequency ranging from 25% to 70%, which in comparison, is described in only 15–20% of patients with MM^2,10,12,27^.

## Prognosis

As in MM, the presence of adverse cytogenetic abnormalities is a major determinant of worse prognosis. Age ≥60 years, platelet count ≤100 × 10^9/^L, and PB PC count ≥20 × 10^9^/L have also been reported as predictors of inferior survival^28^. Historically, patients with pPCL have a median overall survival (OS) ranging from 4 to 11 months^12,29^. Widespread use of novel agents and autologous stem cell transplantation (ASCT) have modestly improved survival. This is illustrated by SEER data reporting median OS of 5, 6, 4, and 12 months in 445 patients diagnosed with pPCL during 1973–1995, 1996–2000, 2001–2005, and 2006–2009, respectively^30^. In patients undergoing ASCT, survival may be 2–3 years, which is still inferior and only a fraction of what can now be seen in other MM populations^31–33^.

## Diagnostic evaluation

The initial diagnostic workup of pPCL is similar to those performed in MM. This includes a CBC/differential, creatinine, calcium, LDH, β2-microglobulin, albumin, serum protein electrophoresis/immunofixation, serum-free light chains, 24 h urine collection for total protein, electrophoresis/immunofixation, and BM aspiration and biopsy^34^. In addition, a PET-CT should be obtained due to the high incidence of extraosseous plasmacytomas. BM specimens should be sent for morphology, flow cytometry, and cytogenetics by FISH^35^. Laboratory tests to assess tumor lysis syndrome (TLS) should also be obtained^35^. A lumbar puncture for cerebrospinal fluid cytology and flow cytometry can be performed, if there is any suspicion for central nervous system or leptomeningeal involvement based on clinical presentation (e.g., headache, cranial nerve palsies, marked PB leukocytosis, and large plasmacytomas appearing to encroach the brain or spinal cord on radiographs).

## Treatment

**Fig. 1:**
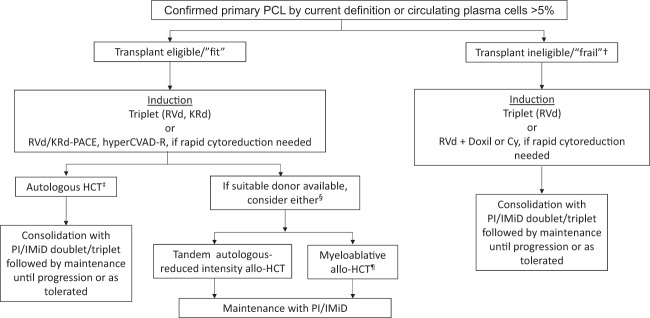
Proposed treatment algorithim for plasma cell leukemia.

Due to the aggressive nature of pPCL, immediate disease control is warranted to prevent disease-related complications and early mortality. Patients with detectable circulating PCs by conventional blood count, despite not meeting the arbitrary cutoff of 20%, should be considered for treatment similar to pPCL. Because there have been no randomized prospective trials that specifically evaluate the treatment of pPCL, therapeutic recommendations are largely based on small prospective and retrospective studies, and extrapolated data from MM trials. Importantly, enrollment to clinical trials is strongly encouraged, especially those incorporating monoclonal antibodies (mAbs) and also other targeted agents (e.g., venetoclax). Figure 1 summarizes a proposed treatment algorithm for pPCL.

### Induction

Regimens that comprise the proteasome inhibitor (PI), bortezomib induce high overall response rates (ORR) of up to 79% in pPCL as demonstrated in retrospective studies^28,36,37^. A phase II study by the IFM demonstrated an ORR of 69% (*n* = 27) after induction with alternating cycles of bortezomib, dexamethasone + cyclophosphamide, or doxorubicin among newly diagnosed patients with pPCL^33^. As in MM, bortezomib may partially abrogate the adverse prognosis of high-risk cytogenetic abnormalities^38,39^, commonly seen in pPCL.

Among the immunomodulatory drugs (IMiDs), lenalidomide is the agent with most data demonstrating efficacy in pPCL. A phase II multicenter study showed an ORR of 60% among patients treated with lenalidomide + dexamethasone with/without high-dose melphalan supported by ASCT^40^. Importantly, next-generation PIs (carfilzomib, ixazomib), IMiDs (pomalidomide), and CD38-directed mAb (daratumumab) have exhibited high efficacy in MM, even among those with adverse cytogenetics. It may be reasonable to speculate that these drugs would have efficacy in pPCL. However, the data on the efficacy of these drugs is exceedingly limited as patients with pPCL are often excluded from prospective trials of MM. Recently, the European Myeloma Network presented an abstract reporting an interim analysis of a phase II trial (EMN12/HOVON 129) using carfilzomib, lenalidomide, and dexamethasone (KRd) as induction, consolidation, and maintenance in patients with pPCL^35^. In patients ≤65 years, 4 cycles of KRd is followed by tandem ASCT (or tandem auto-allo SCT if there is a suitable donor), KRd consolidation, followed by KR maintenance until progression. Patients >65 years old received 8 cycles of KRd followed by KR. Among 14 patients who received 4 cycles of KRd induction, very good partial response or greater response was observed in 80% with 33% achieving at least a complete response. The study suggests that the induction phase could be optimized by incorporating newer agents used in MM to further improve the quality of responses.

For the above reasons, combination therapies incorporating a PI and IMiD are considered frontline in the treatment of pPCL, building on the success of the lenalidomide, bortezomib, and dexamethasone platform (RVd)^41,42^. In patients who are transplant-eligible, induction therapy should be given until best response is achieved. In those who are transplant-ineligible, between 8 and 12 cycles of induction should be administered prior to maintenance therapy.

Although limited evidence exists, intensive chemotherapy regimens, such as VTd/RVd-PACE (bortezomib, thalidomide or lenalidomide, dexamethasone, cisplatin, doxorubicin, cyclophosphamide, etoposide) or hyperCVAD-RV (cyclophosphamide, vincristine, doxorubicin, dexamethasone, lenalidomide, bortezomib) can be used for induction if rapid cytoreduction is required^2,43–45^. Less-intensive regimens such as RVd + liposomal doxorubicin or RVd + cyclophosphamide^46,47^ are also reasonable options to be considered, particularly in frail patients^48^.

Novel MM therapies that are under investigation in pPCL include combination of pomalidomide with ixazomib (NCT02547662), and daratumumab combined with bortezomib, dexamethasone, pegylated liposomal doxorubicin, and lenalidomide (NCT03591744). The remarkable efficacy of the RVd + daratumumab quadruplet as induction therapy achieving deep responses in MM in the GRIFFIN trial strongly advocates for this approach in pPCL, as does the preliminary data with KRd + daratumumab^49,50^. Finally, encouraging data using venetoclax targeting t(11;14) MM provides a clear rationale for incorporating this approach in pPCL, with clinical trials in development for combinations in the newly diagnosed setting^51,52^.

### Hematopoietic cell transplantation

Phase II trials have demonstrated that PI and IMiDs combined with ASCT may be effective in a proportion of patients with pPCL^33,40^. The feasibility and potential efficacy of ASCT in pPCL are also demonstrated in registry studies. The European Group for Blood and Marrow Transplantation reported outcomes of 272 patients with pPCL who underwent ASCT between 1980 and 2006. The median progression-free survival (PFS) and median OS were 14.3 months and 15.7 months, respectively. Data from the Center for International Blood and Marrow Transplant Research (CIBMTR) demonstrated a 3 year PFS and 3 year OS of 34% and 64%, respectively, among 97 patients with pPCL who underwent ASCT^32^. Furthermore, there was a trend toward superior OS in patients who underwent a tandem compared to a single ASCT^32^. Recent data from the CIBMTR demonstrated persistently poor post-ASCT outcomes in 348 patients with pPCL despite the widespread use of novel agents. The 3-year OS was 35% and 38% in patients after ASCT and allo-SCT, respectively^53^. Despite the potential benefit of ASCT, it is clear that intensification of therapy with novel combination regimens in conjunction with ASCT, if appropriate, is still needed to improve pPCL outcomes, as well as innovative approaches to maintenance therapy. One way to intensify treatment is with tandem ASCT, which although largely unproven in pPCL may improve outcomes as demonstrated by better PFS in patients with high-risk MM in the EMN02/HO95^54^ and updated results of the STaMINA trial^55^.

Despite the possibility of cure, there have been variable reports on the efficacy of allo-SCT in pPCL. The CIBMTR reported the outcomes of 50 patients with pPCL who underwent an allo-SCT, the majority of whom (68%) received a myeloablative conditioning regimen^32^. The cumulative incidence of relapse at 3 years was significantly lower with allo-SCT (38% vs. 61%) compared with ASCT but the 3-year OS was inferior with allo-SCT (39% vs. 64%)^32^. The lack of OS benefit with allo-SCT could partly be due to a higher treatment-related mortality (TRM) (41% allo-SCT vs. 5% in the ASCT)^32^. Survival of patients treated with allo-SCT plateaued at 20% in 5 years suggesting that a proportion of patients with pPCL can achieve long-term remissions with this approach. In the IFM trial, 16 responding patients ≤65 years with an HLA-matched donor received a tandem ASCT and reduced-intensity conditioning allo-SCT. Six patients who did not meet criteria for allo-SCT proceeded to a second ASCT followed by consolidation/maintenance with RVd for 1 year. After a median follow-up of 28.7 months, the median PFS and OS were 15.1 and 36.3 months, respectively^33^.

Because allo-SCT is associated with dismal OS when applied after relapse, it should be offered early, if considered at all^56^. As demonstrated in patients with MM, allo-SCT is also fraught by high rates of TRM, particularly with myeloablative conditioning regimens^57,58^. Less-intensive conditioning regimens for allo-SCT that rely on graft versus myeloma (GvM) effects for disease eradication, significantly reduce TRM but at the expense of higher relapse rates^59^. Myeloablative ASCT followed by reduced-intensity allo-SCT (tandem auto/allo-SCT) allows for optimal cytoreduction followed by the immunologic GvM, and in the context of protocol-directed therapy, warrants consideration as part of prospective studies.

### Maintenance

pPCL is characterized by short remissions and early relapse; therefore, early institution of maintenance therapy and/or consolidation post-ASCT (~day 60–80) is recommended to prevent disease progression. Single-agent maintenance appears insufficient to maintain remissions in pPCL as demonstrated by 80% relapse rate occurring at 2–12 months of lenalidomide maintenance^40^. Growing evidence indicates that consolidation with a doublet or triplet may improve response rates and PFS in MM^60^, which may support similar strategies in pPCL such as lenalidomide combined with bortezomib, with or without a mAb.

Maintenance therapy after allo-SCT is controversial but reasonable to administer while awaiting full GvM effect. Lenalidomide may potentiate acute graft-versus host-disease (GvHD) when instituted early after allo-SCT, but may be effective at a lower dose^61,62^. On the other hand, bortezomib is safe and feasible when administered post-allo-SCT^63^, and may decrease the risk of GvHD, partly by impairing the activation of T-cells and antigen-presenting cells^64,65^.

### Supportive care

During treatment initiation, patients with pPCL are at risk for TLS due to a high tumor burden and rapid cell turnover. TLS precautions should be instituted with adequate hydration and prophylaxis with allopurinol or rasburicase (if high-risk). For patients on PIs, varicella zoster prophylaxis should be administered concurrently. Patients should receive venous thromboembolism prophylaxis while on IMiDs. Although osteolytic lesions are less frequent in pPCL relative to MM, all patients with pPCL should be started on anti-resorptive bone targeting agents to reduce the risk of skeletal-related events. Hydration to preserve renal function is also critical, as is more broadly, caution regarding increased risk of infections with particular attention to growth factor use, IVIG administration, and antibiotics as clinically indicated.

### Emerging therapies for PCL

As mentioned, t(11;14) is frequently observed in pPCL and is a marker of BCL-2 overexpression; thus, venetoclax (BCL-2 inhibitor) may be useful. In phase I trials of patients with relapsed/refractory MM, venetoclax has demonstrated high responses as a single agent (ORR 40%)^66^ and in combination with bortezomib and dexamethasone (ORR 78%)^67^ in patients harboring t(11;14). In patients with relapsed pPCL, case reports have demonstrated efficacy of venetoclax as a single agent^68^ or in combination with daratumumab, dexamethasone with^69^ or without bortezomib^70^.

Chimeric antigen receptor (CAR) T-cell therapy^71^ and other immunotherapies (e.g., bispecific T-cell engagers^72^, antibody–drug conjugates^73^) that target B-cell maturation antigen (BCMA) have gained momentum in the recent years due to striking initial responses even among high-risk and heavily pretreated individuals with MM. However, the ability to generate durable functional anti-myeloma T-cell responses is still limited. In a phase I study of bb2121 (BCMA-targeted CAR T-cell), the ORR was 85% among 33 treated patients, including 15 patients with complete responses with median PFS of 11.8 months^71^. The role of CAR T-cells and other immunotherapeutic strategies in the treatment of pPCL remains to be defined.

Other emerging therapies for pPCL include a phase II trial of NK cells in combination with elotuzumab, lenalidomide, and high-dose melphalan before ASCT (NCT01729091) and a phase II trial of panobinostat, gemcitabine, busulfan, and melphalan before ASCT (NCT02506959). In this context, the use of panobinostat in combination with other novel agents including mAbs has a strong rationale; as does the incorporation of selinexor into multiagent regimens, especially given the promising activity of this next-generation novel drug in targeting del(17p)^74,75^.

### Assessing treatment response

In contrast to MM, there are no specific treatment response criteria for PCL. The response criteria used in MM appears inadequate to assess response in PCL due to the leukemic nature of the disease, increased incidence of light chain and nonsecretory forms, and extramedullary disease. The IMWG proposed the inclusion of PB PC criterion and assessment of extramedullary disease (by PET-CT) to the standard biomarker and BM criteria (Table 1)^2^. In case of undetectable BM and PB PCs by morphology, flow cytometry should be used to measure residual disease.

**Table 1:** Plasma cell leukemia response criteria by the International Myeloma Working Group.

Response	Serum and urine biomarkers^a^	BM plasma cells	Peripheral blood plasma cells	Extramedullary disease
Stringent complete response (CR)	Negative serum & urine immunofixation; Normal serum-free light chain ratio	<5% by morphology & no malignant plasma cells by flow cytometry	Negative by morphology & flow cytometry	Absent
Complete response	Negative serum & urine immunofixation^b^	<5% by morphology	Negative by morphology	Absent
Very good partial response	≥90% reduction of serum M-protein & <100 mg/24 h urinary M-protein^c^	<5% by morphology	Negative by morphology	Absent
Partial response	≥50% reduction of serum M-protein & reduction in 24 h urinary M-protein by ≥90% & <200 mg/24 h^d^	5–25% by morphology	1–5% plasma cells by morphology	≥50% size reduction from baseline
Stable disease	Not meeting the criteria of either partial response or progressive disease			
Progressive disease	>25% increase in the level of the serum M-protein with an absolute increase ≥5 g/L;>25% increase in the 24 h urinary light chain excretion with an absolute increase ≥200 mg/24 h	>25% increase or absolute increase ≥10%	>5% absolute increase in plasma cells by morphology	Increase in size or number
Relapse from CR	Reappearance of original M-protein in serum and/or urine immunofixation	>10% increase	Detectable at any level	Any extramedullary disease

^a^It should be maintained for a minimum of 6 weeks. In case of discrepancy or undetectable serological parameter, the patient must be classified according to bone marrow criteria.

^b^If the serum and urine M-protein are unmeasurable, a normal serum kappa/lambda ratio is also required.

^c^If the serum and urine M-protein are unmeasurable, a ≥90% decrease in the difference between involved and uninvolved light chains is required.

^d^If the serum and urine M-protein are unmeasurable, a ≥50% decrease in the difference between involved and uninvolved light chains is required.

### Treatment of relapsed or refractory PCL, including secondary PCL

Limited data exists to guide treatment for relapsed/refractory pPCL. Clinical trial participation should be encouraged if an appropriate study is available; although pPCL is typically an exclusive criteria to most studies in the advanced disease setting. A critical option is to utilize a combination of active drugs in MM, particularly ones that the patient has not previously received nor is refractory to. Patients who experience a deep and prolonged response to a prior therapy, re-treatment with the same regimen can be considered, particularly if the relapse occurred off therapy^76^.

For patients in whom RVd-based therapy has been utilized initially, carfilzomib-based treatment can be deployed at relapse; similarly, if MM disease progression manifesting as secondary PCL occurs, the use of carfilzomib-based therapy should also be used in the same context. This sequencing of therapeutic strategies has been recently validated by the events of the ENDURANCE trial comparing RVd to KRd in the standard newly diagnosed MM patient where equivalent outcomes and less cardiovascular, renal, and pulmonary toxicity was seen with RVd^77^.

Salvage strategies using combinations of novel agents incorporating pomalidomide, panobinostat, elotuzumab, daratumumab, isatuximab, and if appropriate ixazomib, selinexor, and belantamab mafodotin can all be considered^78–85^. Combination regimens incorporating chemotherapy are also a potentially mainstay in this frequently difficult-to-treat population, and the use of high-dose steroids as well as nonmyelotoxic agents such as thalidomide as part of these approaches can have merit^86,87^.

It is hoped that additional approaches can be engendered from the next wave of novel agents, and especially those targeting “stemness” and extramedullary disease, such as melflufen, with current studies informing real-world practice^88–90^.

## Conclusion

The use of multidrug combinations (including a PI, an IMiD, and now a mAb) for induction appears to be a rational approach to consider in pPCL. While rates of post-ASCT relapse remain high, ASCT should be incorporated in eligible patients when appropriate, either as tandem ASCT or tandem ASCT/allo-SCT, comparable to the paradigm used more broadly in relapsed/refractory MM, given the need for sustained response. Similarly, because of the high TRM, myeloablative allo-SCT should only be performed in the context of a trial. Consolidation and maintenance until disease progression should be administered to both transplant-eligible and ineligible patients. Although enrollment to clinical trials is strongly advised, this is not always feasible partly due to the relative paucity of specific studies on pPCL, highlighting the critical need for prospective trials for this especially challenging high-risk subgroup. The role of immunotherapies (CAR T-cells, mAbs, bispecific T-cell engagers, antibody–drug conjugates) and small molecule inhibitors, such as venetoclax, in the treatment of pPCL is much needed and eagerly awaited.
